# Supplementary material for: Large oscillatory thermal hall effect in kagome metals
Source: Nat Commun. 2024 Jul 23;15:6224. doi: 10.1038/s41467-024-50336-7 (PMC11266402; doi:10.1038/s41467-024-50336-7)
Supplement: Supplementary file 1 — Supplementary Information [file 41467_2024_50336_MOESM1_ESM.pdf]

Supplementary Information for

Large Oscillatory Thermal Hall Effect in Kagome Metals

This PDF file includes:

Supplementary Note

1. Measuring the thermal resistivity and conductivity

2. Impact of Phonon on the total thermal conductivity and analysis of quantum oscillation of the thermal transport property

3. Excluding the phonon contribution to the oscillatory component of κ_{xy}

4. Excluding artifact for the oscillation of λ_{xy}

5. Quantum Oscillation for Thermal Transport Properties

6. Band pass filter

7. Definition and calculation of the phase sharpness parameter ψ

8. Hall angle determined by the quantum oscillations

Supplementary Figure 1 to 13

Supplementary References

Supplementary Note 1. Measuring the thermal resistivity and conductivity

In linear response, the charge and heat current densities J_e and J_q are given by the Onsager relations

$$\begin{aligned} J_e &= \sigma E - \alpha \nabla T \\ J_q &= \alpha T E - \kappa \nabla T \end{aligned} \tag{1}$$

Here, α is the Peltier-Ettingshausen coefficient. In the thermal conductivity measurement, there is no J_e, then J_q can be written as follows when only a temperature gradient is present

$$J_q = \left(\frac{\alpha^2 T}{\sigma} - \kappa \right) \nabla T \tag{2}$$

Since the electric conductivity σ is very high, the $-\frac{\alpha^2 T}{\sigma}$ term is negligible compared to κ. The thermal conductivity and thermal Hall coefficient can be obtained by measuring the $x-y$ plane temperature gradient $-\nabla_j T$ under a constant thermal current density J_{qi} produced by the heater

$$J_{qi} = -k_{ij} \nabla_j T \tag{3}$$

The thermal resistivity matrix λ_{ji} satisfies the relation

$$\lambda_{ji} J_{qi} = -\nabla_j T \tag{4}$$

Since J_{qi} is a constant, λ_{ji} is directly proportional to the measured $x-y$ plane temperature gradient $-\nabla_i T$, $-\nabla_j T$ and J_{qi} can be calculated using:

$$\begin{aligned} -\nabla_j T &= \Delta T_j / l_j \\ J_{qi} &= P / (l_y \cdot l_z) \end{aligned} \tag{5}$$

where ΔT_i is the longitudinal or transverse temperature difference, l_j is the distance between the contacts on the sample, l_y is the sample width, and l_z is the sample thickness. Ideally, the thermal current should be along the longitudinal and perpendicular to the transverse direction, so only J_{qx} is present. From the raw data, ΔT_x is almost symmetric under H. However, due to the contact misalignment, ΔT_y isn't fully H-antisymmetric and always contains the longitudinal pick-up. So, the field anti-symmetrization is applied to extract only the Hall component. After λ_{ij} is obtained, the thermal conductivity matrix can be resolved by doing the matrix inversion, that is,

$$\kappa_{ij} = (\lambda^{-1})_{ij} \tag{6}$$

Supplementary Note 2. Impact of Phonon on the total thermal conductivity and analysis of quantum oscillation of the thermal transport property

The thermal conductivity κ_{xx} of CsV_3Sb_5 violates the WF law even down to the lowest T. Due to a significant phononic contribution, κ_{xx} can be described as a sum of the electron and the phonon thermal conductivity:

$$\kappa_{xx} = \kappa_e + \kappa_{ph} \tag{7}$$

Here, κ_e comes from the electrons and κ_{ph} is the phonon thermal conductivity. Experimentally, we found the thermal Hall angle $\tan\theta_H$, which is defined as the ratio of κ_{xy} and κ_{xx}, increases as T goes lower, as shown in Fig. S3. According to the WF law, $\kappa_e = L_0\sigma_{xx}T$, and $\kappa_{xy} = \tan\theta_e\kappa_e$. The phononic part κ_{ph} can be expressed as CT^β, where C is a constant. Then, $\tan\theta_H$ is given by:

$$\tan\theta_H = \frac{\tan\theta_e\kappa_e}{\kappa_e + CT^\beta}$$

Since below $\sim$ 5 K, $\tan\theta_e$ is nearly a constant 0.21, and σ_{xx} can also be assumed to be constant. By fitting the T dependence of $\tan\theta_H$ at H = 13 T, C is found to be $0.85L_0\sigma_{xx}$, and β is 1.68. Furthermore, using the obtained parameters, the phononic contribution $0.85L_0\sigma_{xx}T^{1.68}$ is consistent with the measured κ_{xx} at H = 13 T.

Because phonons are bosons, they should not establish quantum oscillations. We note, however, that the existence of the phonon thermal conductivity may affect the observed quantum oscillation amplitude in the thermal transport properties. Since κ_{xx} is a sum of κ_e and κ_{ph}, the oscillatory component of κ_{xx} comes from κ_e. The temperature gradient, proportional to the thermal resistivity matrix, is detected directly during the measurement. To resolve the temperature dependence of the oscillatory components from the electron, κ_{ph} needs to be taken into consideration. In other words, with κ_{ph} in presence, the temperature dependence of the oscillation amplitude of λ_{ij} differs from the second derivative of R_T. For the longitudinal one, if we make use of the fact that the thermal Hall angle is small, then λ_{xx} is inversely proportional to κ_{xx}, that is

$$\nabla T_{xx} \propto \frac{1}{\widetilde{\kappa}_e + \overline{\kappa}_e + \kappa_{ph}} \tag{8}$$

Thus, the oscillatory part $\nabla\widetilde{T}_{xx}$ can be obtained by

$$\frac{\nabla\widetilde{T}_{xx}}{\nabla T_{xx}} = \frac{\widetilde{\kappa}_e}{\overline{\kappa}_e + \kappa_{ph}} \tag{9}$$

That means

$$\frac{\widetilde{\lambda_{xx}}}{\lambda_{xx}^2} = \widetilde{\kappa_e} = \widetilde{\kappa_{xx}} \tag{10}$$

Due to the phonon contribution, λ_{xx} becomes smaller than the electronic thermal resistivity. As a result, the oscillation in λ_{xx} is weakened.

For the transverse thermal resistivity, the oscillaiton is much more complicated when considering the phonon as well as the oscillation in κ_{xx}, since λ_{xy} is calculated using

$$\lambda_{xy} = \frac{\kappa_{xy}}{\kappa_{xx}^2 + \kappa_{xy}^2} \tag{11}$$

If we simply ignore the impact of the oscillations in κ_{xx} to that in $\widetilde{\lambda_{xy}}$, and also assume the thermal Hall angle is small, then the oscillations in the transverse channel obey the same relation as $\widetilde{\lambda_{xx}}$, which is

$$\frac{\widetilde{\lambda_{xy}}}{\lambda_{xx}^2} = \widetilde{\kappa_{xy}} \tag{12}$$

That means the exact temperature dependence of $\Delta\lambda_{xx}$ and $\Delta\lambda_{xy}$ deviate from the second derivative of R_T. The λ_{xx}^2 background needs to be considered when the oscillation amplitude is compared with $R_T''(X)$.

Moreover, in the idealized case, the thermal Hall measurement should be done when the temperature in the middle of the sample keeps constant, and the temperature gradient maintains the same value. However, due to the limitation of the experimental setup, only one end of the sample can be thermally anchored to the heat bath. Thus, the thermal Hall signal is detected at an oscillatory temperature gradient as the heat capacity oscillates with the magnetic field.

Supplementary Note 3. Excluding the phonon contribution to the oscillatory component of κ_{xy}

While the phonon contribution is observed to enhance the quantum oscillation amplitude in bulk antimony[1], the phonon contribution is unlikely to the origin of the large enhancement in the oscillatory component of κ_{xy} in CsV_3Sb_5.

1. Phonon has only been discovered to enhance the longitudinal thermal conductivity, such as the case of antimony. However, phonon has not been observed to contribute to any oscillations in the transverse thermal Hall conductivity. In CsV_3Sb_5, it is much less likely because phonon does not even contribute to the non-oscillating thermal Hall background signal.

2. Phonon drag effect was not observed in CsV_3Sb_5 from the thermoelectric measurements[2,3]. Moreover, from the transverse WF law, phonon does not even contribute to the thermal Hall effect in CsV_3Sb_5. Thus, the oscillations of the thermal Hall conductivity in CsV_3Sb_5 are free of phonon contribution.

3. The electron-phonon bifluid behavior was recently observed in bulk antimony, and the quantum oscillation of thermal conductivity contains phonon contribution[1]. The strong electron-phonon coupling in antimony leads to the formation of electron-phonon bifluid, thus the thermal conductivity oscillates. Showing in Fig. 4a of the reference[1], as the temperature is getting closer to the base temperature, the oscillation amplitude $\delta\kappa/\kappa_{\mathrm{ph}}$ becomes smaller, which is clearly the behavior of the phonon contribution. However, the case in CsV_3Sb_5 is totally different. In CsV_3Sb_5, $\delta\kappa_{xy}/\kappa_{\mathrm{xy}}$ becomes a constant near the base temperature, which confirms all the contribution to the oscillation is from the electron, for both longitudinal and transverse channel.

4. From our experimental results, we did detailed analysis of the temperature dependence of the oscillation amplitude. We found the oscillation amplitude in both κ_{xx} and κ_{xx} obeys the second derivative of the LK formula after properly considering the influence of the phonon thermal conductivity to the κ_{xx} and κ_{xx} oscillation amplitude. (The analysis can be found in the Supplementary Note 2: Impact of Phonon on the total thermal conductivity and analysis of quantum oscillation of the thermal transport property. However, phonon thermal conductivity does not display any oscillations) This particular temperature dependence is an essential signature of the Fermionic quasiparticles.

5. When the temperature is low enough and the temperature smearing effect of the quantum oscillation is becoming weak, the oscillation amplitude of both κ_{xx} and κ_{xx} over temperature becomes nearly constant all the way down to the base temperature, which is demonstrated as Fig. 3b in our manuscript. If any phonon contributes to the oscillation, the oscillation amplitude over temperature would continue to decrease when temperature is getting lower.

Given these five clear evidences, we think we can exclude the contribution of phonons to quantum oscillations in the thermal Hall effects.

Supplementary Note 4. Excluding artifact for the oscillation of λ_{xy}

Due to the contact misalignment, the thermal Hall signal is extracted by performing the field antisymmetrization. Moreover, the oscillations in λ_{xx} can induce the oscillation of the sample average

temperature T_S, although the change of T_S is always kept being less than 10 % of the heat bath temperature and the oscillation amplitude of the λ_{xx} is much smaller compared to the magnetothermal conductivity background signal. To confirm the measured oscillation is intrinsic, not from the data analysis process or longitudinal oscillation pick-up, the raw data of the longitudinal and transverse temperature differences at 1.04 K is shown in Fig. S4a. In Fig. S4b, the transverse T differences at $H > 0$ and $H < 0$ are plotted together as a function of $|H|$. Although the longitudinal pickup is unavoidable, the opposite phase of $H > 0$ and $H < 0$ data can be seen when $|H|$ is greater than 8 T. Possible extrinsic effects can never produce such a phase reversal.

Supplementary Note 5. Quantum Oscillation for Thermal Transport Properties

5.1. The mean free path approximation

Under the semiclassical theory, the mean free path approximation gives:

$$\kappa_{xx} = \frac{1}{3} n \langle v \rangle \bar{\lambda} C \tag{13}$$

$$\kappa_{xy} = \kappa_{xx} \tan\theta_H = \kappa_{xx} \omega_c \tau \tag{14}$$

Here, C is the heat capacity of the charge carriers, n is the density of the carriers, $\langle v \rangle$ is the mean velocity, $\overline{\lambda}$ is the mean free path, θ_H is the thermal Hall angle, ω_c is the cyclotron frequency of the fermion, and τ is the scattering time. The oscillatory component of both κ_{xx} and κ_{xy} comes from C, that is, $\Delta\tilde{\kappa} \propto \Delta\tilde{C}$. To estimate ΔC at constant T, the thermodynamic potential Ω can first be defined as:

$$\Omega = U - TS - N\varepsilon_F \tag{15}$$

where U is the internal energy, S is the entropy of the system, N is the number of fermions, and ε_F is the Fermi energy. By differentiating Ω and performing the Legendre transformation:

$$\mathrm{d}\Omega = -SdT - MdH - Nd\varepsilon_F \tag{16}$$

Thus, the entropy S can be calculated using:

$$S = -(\partial\Omega/\partial T)_{H,\varepsilon_F} \tag{17}$$

The heat capacity is:

$$C = T(\partial \mathrm{S}/\partial \mathrm{T})_{H,N} \tag{18}$$

Therefore, the oscillatory part of the heat capacity $\Delta\tilde{C}$ is given by:

$$\Delta\tilde{C} = T(\partial\tilde{\mathrm{S}}/\partial\mathrm{T})_{H,N} = -T\left[\frac{\partial}{\partial\mathrm{T}}\left(\frac{\partial\widetilde{\Omega}}{\partial\mathrm{T}}\right)_{H,\varepsilon_F}\right]_{H,N} \tag{19}$$

Quantum oscillations are the result of Landau Level quantization for Fermi surfaces. The thermodynamic potential $\widetilde{\Omega}$ Can be expressed as[4]:

$$\widetilde{\Omega} = A\sum_{p=1}\frac{1}{p^{\frac{5}{2}}}\cos\left(2\pi p\frac{F}{H}+\phi\right) \tag{20}$$

where A is the amplitude, p is the harmonic content, F is the dHvA oscillation frequency, and ϕ is the phase. In practical cases, the finite temperature, finite electron relaxation time, and electron spin can all be regarded as the phase smearing factor. The phase is varied over a small range around the value corresponding to the idealized situation. Among these three damping factors, only the temperature damping effect can provide a factor with a non-zero derivative w.r.t T. At finite T, since the energy ε spreads around ε_F obeying the Fermi-Dirac distribution, and the frequency F is a function of ε. Thus, the spreading of ε leads to the smearing of the phase, and the amplitude of the oscillatory content in $\widetilde{\Omega}$ is reduced by a factor R_T, which is already known as:

$$R_T = \frac{2\pi^2 pkT}{\hbar\omega_c}/\sinh\left(\frac{2\pi^2 pkT}{\hbar\omega_c}\right) \tag{21}$$

where $\omega_c = \frac{eB}{m^*}$, p is an integer representing the harmonic content. The reduction factor R_T greatly reduces the harmonic content of oscillation, so it's a good approximation to write:

$$\widetilde{\Omega} = \widetilde{\Omega}_0 R_T(T) = \widetilde{\Omega}_0\frac{2\pi^2 kT}{\hbar\omega_c}/\sinh\left(\frac{2\pi^2 kT}{\hbar\omega_c}\right) \tag{22}$$

where the subscript 0 in $\widetilde{\Omega}_0$ stands for the oscillatory component at T = 0 K. Now we insert the equation above to Supplementary Eq. (20), the oscillations in the heat capacity $\Delta\tilde{C}$ can be calculated by taking the second derivative with respect to T:

$$\Delta\tilde{C} = -T\widetilde{\Omega}_0\frac{d^2}{dT^2}R_T(T) \tag{23}$$

Then, the oscillatory part of the thermal Hall conductivity $\Delta\widetilde{\kappa}_{xy}$ is given by:

$$\Delta\widetilde{\kappa}_{xy} = \frac{1}{3}n\langle v\rangle\bar{\lambda}\,\omega_c\tau\Delta\tilde{C} = -\frac{1}{3}n\langle v\rangle\bar{\lambda}\,\omega_c\tau T\widetilde{\Omega}_0\frac{d^2}{dT^2}R_T(T) \tag{24}$$

Therefore, the oscillation in the thermal Hall conductivity due to the Landau Level quantization only comes from oscillatory part of the thermodynamic potential. Most importantly, the temperature dependence of the oscillation amplitude in the thermal Hall conductivity is determined by the second derivative of the R_T term in the LK formula, i.e.

$$\frac{\Delta\widetilde{\kappa}_{xy}}{T} = \left(\frac{\Delta\widetilde{\kappa}_{xy}}{T}\right)_{T=0} \frac{R_T''(T)}{R_T''(T=0)} \tag{25}$$

We note the oscillation amplitude for both $\Delta\lambda_{xx}$ and $\Delta\lambda_{xy}$ shows a concave curve that does not exactly follow the second derivative of $R_T(T)$. We attribute this deviation to the non-oscillating phonon contribution to the thermal conductivity.

5.2. Boltzmann theory

The second derivative relation for the oscillation in κ_{xx} and κ_{xy} can also be derived following the Boltzmann theory. Under Boltzmann[5] or Landauer[6] formalism, the conductivity and thermal conductivity tensor can be calculated using:

$$\sigma = \frac{2e^2}{h}\int D(\varepsilon)\sigma(\varepsilon)\mathrm{d}\varepsilon \tag{26}$$

$$\kappa = -\frac{2e^2T}{h}\left(\frac{k_B}{e}\right)^2\int\left(\frac{\varepsilon-\varepsilon_\mathrm{F}}{k_BT}\right)^2 D(\varepsilon)\sigma(\varepsilon)\mathrm{d}\varepsilon \tag{27}$$

where h is the Planck constant, $D(\varepsilon) = -\frac{\partial f}{\partial \varepsilon}$ is the derivative of Fermi-Dirac distribution function, $\boldsymbol{\sigma}(\varepsilon)$ is the electrical conductivity tensor of energy ε. $\boldsymbol{\sigma}$ and κ are the electrical and thermal conductivity tensors, respectively. The oscillatory components of the electrical and thermal conductivity tensors $\Delta\widetilde{\boldsymbol{\sigma}}$ and $\Delta\widetilde{\boldsymbol{\kappa}}$ can also be obtained by replacing $\boldsymbol{\sigma}(\varepsilon)$ with the oscillatory term $\Delta\widetilde{\boldsymbol{\sigma}}(\varepsilon)$. Microscopically, the change of energy ε corresponds to the change of phase ϕ:

$$\frac{\varepsilon-\varepsilon_F}{kT} = \frac{\hbar\omega_c\phi}{2\pi kT} \equiv \frac{\phi}{\lambda} \tag{28}$$

with λ defined as $2\pi kT/\hbar\omega_c$. The effect of a finite temperature is equivalent to a phase smearing with the $D(\varepsilon)$ distribution. Macroscopically, the state and frequency probed by the quantum oscillation are centered at a given location in the k space. The effect of the phase smearing is to multiply the amplitude by the reduction factor R_T, which can be calculated by performing the Fourier transform to ε:

$$R_T = \left|\int_{-\infty}^{\infty} e^{i\lambda\varepsilon}D(\varepsilon)d\varepsilon\right| / \int_{-\infty}^{\infty} D(\varepsilon)d\varepsilon \tag{29}$$

The integral gives:

$$R_T = \frac{\pi\lambda}{\sinh\pi\lambda} = \frac{2\pi^2kT}{\hbar\omega_c} / \sinh\left(\frac{2\pi^2kT}{\hbar\omega_c}\right) \tag{30}$$

To calculate the reduction factor $R_{T,\ \kappa}$ for the oscillation amplitude of the thermal and thermal Hall conductivity, the distribution function $D(\varepsilon)$ needs to multiply a term $z^2 = (\frac{\varepsilon-\varepsilon_\mathrm{F}}{k_BT})^2$, which is shown in Fig. S7. Then, the reduction factor is obtained by performing the Fourier transform to ε with the equivalent distribution function $z^2 D(\varepsilon)$:

$$R_{T,\kappa} = \mathscr{F}\left(z^2 D(\varepsilon)\right) / \int_{-\infty}^{\infty} z^2 D(\varepsilon) d\varepsilon = \left| \int_{-\infty}^{\infty} \left(\frac{\varepsilon-\varepsilon_\mathrm{F}}{k_BT}\right)^2 D(\varepsilon) e^{i\lambda\varepsilon} d\varepsilon \right| / \int_{-\infty}^{\infty} \left(\frac{\varepsilon-\varepsilon_\mathrm{F}}{k_BT}\right)^2 D(\varepsilon) d\varepsilon \tag{31}$$

The Fourier transformation to the $z^2 D(\varepsilon)$ term is equivalent to calculate the convolution of the Fourier transform of the two functions, which is:

$$\mathscr{F}\left(z^2 D(\varepsilon)\right) = \mathscr{F}\left(z^2\right) * \mathscr{F}(D(\varepsilon)) \tag{32}$$

The first Fourier transform gives the second derivative of the delta function:

$$\mathscr{F}\left(z^2\right) = -\sqrt{2\pi}\delta'' \tag{33}$$

The convolution can then be solved by doing the integration by parts, and the result is:

$$R_{T,\kappa} = T\frac{d^2}{dT^2} R_T(T) / \frac{d^2}{dT^2} R_T(T=0) \tag{34}$$

For the thermal resistivity, under the assumption that $\boldsymbol{\kappa}$ and $\boldsymbol{\lambda}$ are measured isothermally, then $\boldsymbol{\kappa\lambda} = 1$. Since $\bar{\boldsymbol{\kappa}}\bar{\boldsymbol{\lambda}} = 1$, where the bar means the background part other than the oscillatory component, then the relation for the oscillatory thermal conductivity and resistivity tensor is:

$$\overline{\boldsymbol{\kappa}} \Delta\tilde{\boldsymbol{\lambda}} = -\overline{\boldsymbol{\lambda}} \Delta\tilde{\boldsymbol{\kappa}} \tag{35}$$

Thus, For the $\Delta\widetilde{\lambda_{ij}}$, R_T needs to be replaced by $-3TR_T''(X)$. At different H, we extracted the temperature dependence of the oscillation amplitude from the δ orbit and fit it with the second derivative of R_T.

Supplementary Note 6. Band pass filter

The quantum oscillations of κ_{xy} consist of different frequencies from multiple orbits. Here, four principal frequencies, $\mathrm{F}_\alpha = 11$ T, $\mathrm{F}_\beta = 25$ T, $\mathrm{F}_\gamma = 72$ T, $\mathrm{F}_\delta = 87$ T is observed. Depending on their effective mass, the oscillation amplitudes of the four orbits have various reduction factors over T and pass zero at different T and H. Thus, it's important to focus on the contribution from a single orbit. Among all these four compositions, the contribution from the δ orbit (frequency

F∼87 T) with a clear phase dominates when H is greater than ∼8 T. A high-pass filter (HPF) is applied to pass with a frequency higher than 75 T and attenuates signals from α, β, and γ orbits. Fig. S5 shows the oscillation components $\Delta\lambda_{xy}T$ after a fifth-order polynomial background subtraction from the raw data without (Fig. S5a) and with (Fig. S5b) the 75 T HPF. After the HPF is applied, the low-frequency oscillations are successfully attenuated.

Supplementary Note 7. Definition and calculation of the phase sharpness parameter ψ

From the oscillatory components $\Delta\lambda_{xy,\delta}T$ of the δ orbit at lowest T (0.55 K, after the 75 T HPF is applied), the angular frequency ω and the phase φ can be extracted. First, we define the in-phase and out-of-phase reference wave functions I_R and O_R as:

$$I_R = \sin\left(\frac{\omega}{\mu_0 H} + \varphi + \pi/2\right) \tag{36}$$

$$O_R = \sin\left(\frac{\omega}{\mu_0 H} + \varphi - \pi/2\right) \tag{37}$$

”In-phase oscillation” means it is in-phase with the oscillatory components $\Delta\lambda_{xy,\delta}T$ of the δ orbit at base temperature and strong magnetic field. At higher temperature and weaker field, the phase of the oscillation becomes opposite, then it is called “out-of-phase”.

Since the magnetothermal quantum oscillation phase can either be in-phase at lowest T and strong H, or out-of-phase higher T and weaker H, it is a binary variable. We define and calculate the phase sharpness parameter ψ to tell whether the oscillation is in-phase or out-of-phase.

Then, I_R has a unitary amplitude and the same ω and φ with the oscillatory components at lowest T, and the phase difference between O_R and I_R is π. Next, we define the phase sharpness parameter ψ to determine whether the oscillation at various H and elevated T is more like I_R or O_R. It can be expected at lower T, the oscillatory components are more similar to I_R while at elevated T it's the opposite. To quantitively estimate the similarity, we calculate the 2-norm of the difference between the oscillatory components and the reference at each T and period of oscillation in $\frac{1}{\mu_0 H}$:

$$\Delta_I = \sqrt{\int \left| \Delta\lambda_{xy,\delta}\left(\frac{1}{\mu_0 H}\right) T/A_{xy,\delta} - I_R\left(\frac{1}{\mu_0 H}\right)\right|^2 dH} \tag{38}$$

$$\Delta_O = \sqrt{\int \left| \Delta\lambda_{xy,\delta}\left(\frac{1}{\mu_0 H}\right) T/A_{xy,\delta} - I_O\left(\frac{1}{\mu_0 H}\right)\right|^2 dH} \tag{39}$$

where $A_{xy,\delta}$ is the amplitude of $\Delta\lambda_{xy,\delta}T$, and the integration is within each period of oscillation. To define the parameter ψ which shows the phase sharpness, we determine a suitable cutoff ε. If

both Δ_I and Δ_O are greater than ε, $\psi = \Delta_I/\Delta_O$. Otherwise, we set $\psi = 1$. At lower T and stronger H, $\psi > 1$, $\Delta\lambda_{xy,\delta}T$ is in-phase. At higher T and weaker H, $\psi < 1$, the phase becomes opposite. At the phase-shifting boundary, ψ is almost 1.

Supplementary Note 8. Hall angle determined by the quantum oscillations

The validity of the Wiedemann-Franz law for the background of electrical and thermal conductivity requires the electrical transport relaxation time τ_e is equal to the thermal transport relaxation time τ_{th}, which means that the details of the electron scattering processes in electrical transport are the same as that in the thermal transport. For quantum oscillations, the validity of the Wiedemann-Franz law requires that the dHvA and the SdH oscillations have the same quantum relaxation time, which means the oscillatory density of states are from the same Landau level at the same location on the Fermi surface. On the other hand, since QOs usually only capture the fermionic contribution, the ratio of the amplitudes of the oscillatory components $\Delta\kappa_{xy(T\to0)}/\Delta\kappa_{xx(T\to0)}$ or $\Delta\sigma_{xy(T\to0)}/\Delta\sigma_{xx(T\to0)}$ reflect the electrical Hall angle $\omega_c\tau_e$ in the ground state. Experimentally, the thermal Hall conductivity and electrical Hall conductivity background obey the WF law. Fig. S2 shows both the Hall effect and the magnetoresistance of the same crystal. The resulting electrical Hall angle $\omega_c\tau_e$ is ~ 0.21 at base temperature. This value is consistent with the ratio $\Delta\kappa_{xy(T\to0)}/\Delta\kappa_{xx(T\to0)}$ and $\Delta\sigma_{xy(T\to0)}/\Delta\sigma_{xx(T\to0)}$.

However, the experimental data shows enhanced oscillation amplitude in magnetothermal conductivity compared to the electrical conductivity. By calculating the ratio between them, we find $\Delta\kappa_{xy(T\to0)}/\Delta\sigma_{xy(T\to0)}T = 2.50L_0$ and $\Delta\kappa_{xx(T\to0)}/\Delta\sigma_{xx(T\to0)}T = 1.55L_0$. From another perspective, the discrepancy is also evident by comparing the value of $\Delta\kappa_{xy(T\to0)}$ and $\Delta\kappa_{xx(T\to0)}$, and their ratio $\Delta\kappa_{xy(T\to0)}/\Delta\kappa_{xx(T\to0)}$ is $\sim 1.61\omega_c\tau_e$ from the experiments. The above analysis gives these three inequalities:

$$\frac{\Delta\kappa_{xy}}{\kappa_{xy}} > \frac{\Delta\sigma_{xy}}{\sigma_{xy}} \tag{40}$$

$$\frac{\Delta\kappa_e}{\kappa_e} > \frac{\Delta\sigma_{xx}}{\sigma_{xx}} \tag{41}$$

$$\frac{\Delta\kappa_{xy}}{\Delta\kappa_{xx}} > \frac{\Delta\sigma_{xy}}{\Delta\sigma_{xx}} = \frac{\sigma_{xy}}{\sigma_{xx}} = \omega_c\tau_e \tag{42}$$

These inequalities provide robust evidence for the unconventional large enhancement of the thermal Hall oscillations in this Kagome metal.

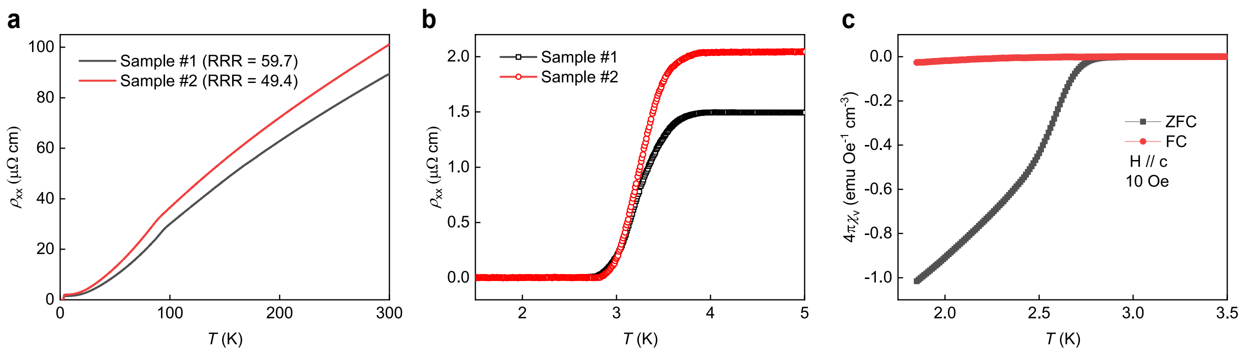

Supplementary Fig. 1. **Temperature dependence of resistivity and magnetization of CsV_3Sb_5 single crystal.** (A, B)Temperature dependence of resistivity of Sample #1 and #2 in the range from 1.5 K to 300 K (Panel A) and from 1.5 K to 5 K (Panel B). (C) The low-field magnetization curve of CsV_3Sb_5 showing the Meissner effect. Source data are provided as a Source Data file.

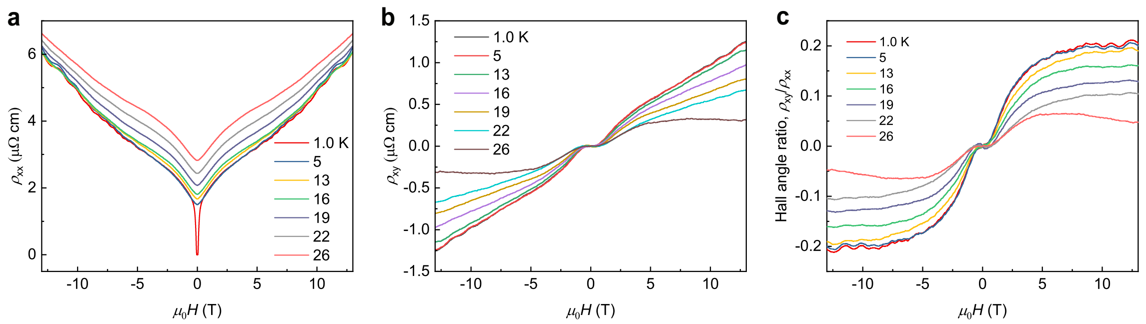

Supplementary Fig. 2. **Electronic oscillation.** Panel (a) shows the magnetoresistance measured at various temperatures with field along *c* axis. The data is measured by the same leads as the magnetothermal measurement setup. Panel (b) shows the electrical hall resistivity measured at various temperatures. The low-field region shows an anomalous Hall effect. Panel (c) shows the *H*-dependence of the electrical Hall angle defined as the ratio between ρ_{xy} and ρ_{xx}. Source data are provided as a Source Data file.

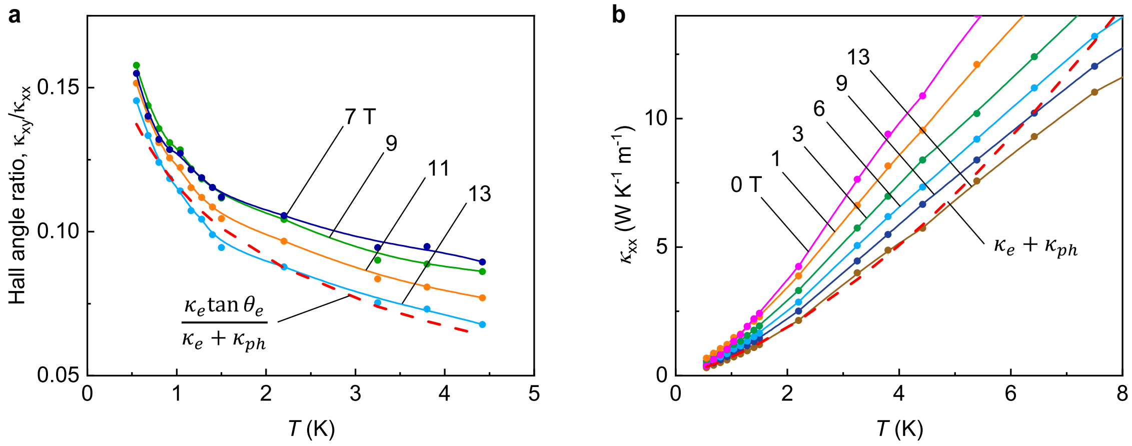

Supplementary Fig. 3. **Temperature variance of $tan\theta_H$ and κ_{xx} with the fitting functions.** Panel (a) shows the temperature variance of the measured $tan\theta_H$ at selected H. The red dashed curve shows the fitting using the function $\tan\theta_H = \frac{\tan\theta_e \kappa_e}{\kappa_e + CT^\beta}$ at H=13T. Panel (b) shows the temperature variance of κ_{xx} at selected H. The red dashed curve shows the function $\kappa_{xx} = \kappa_e + \kappa_{ph} = L_0\sigma_{xx}\left(1 + CT^\beta\right)$, where C and β is obtained from the fitting of $\tan\theta_H$. Source data are provided as a Source Data file.

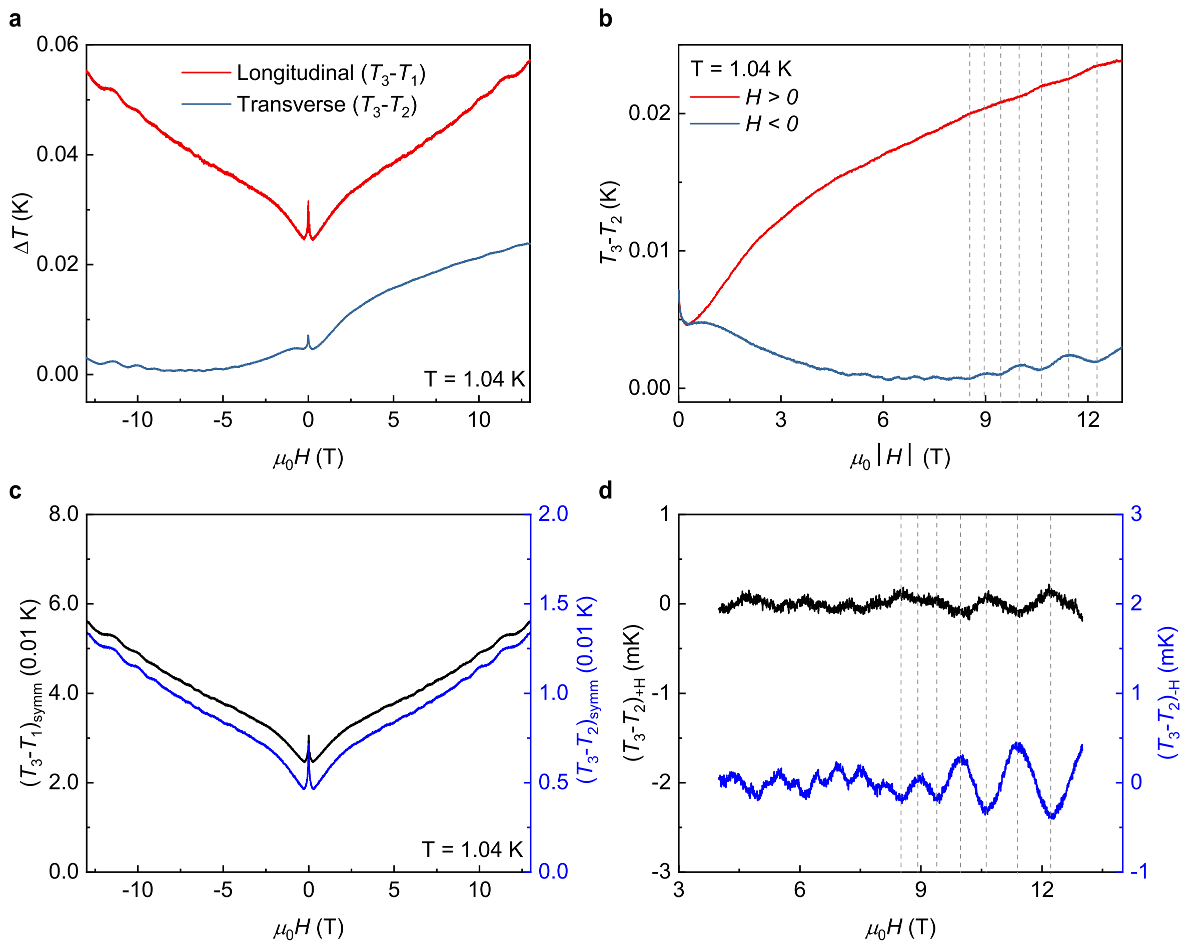

Supplementary Fig. 4. **Excluding artifact for the oscillation of λ_{xy}.** Panel (a) shows the longitudinal and transverse temperature differences measured on the sample at 1.04 K. Panel (b) shows the positive and negative field data of the transverse temperature difference. Panel (d) shows only the oscillatory components after the fifth-order polynomial background subtraction. The peaks and troughs are marked by a grey dashed line. When one curve shows a peak, the other one displays a trough. Panel (c) compares the symmetric component in the longitudinal and transverse temperature differences, and these two curves are closely similar. Source data are provided as a Source Data file.

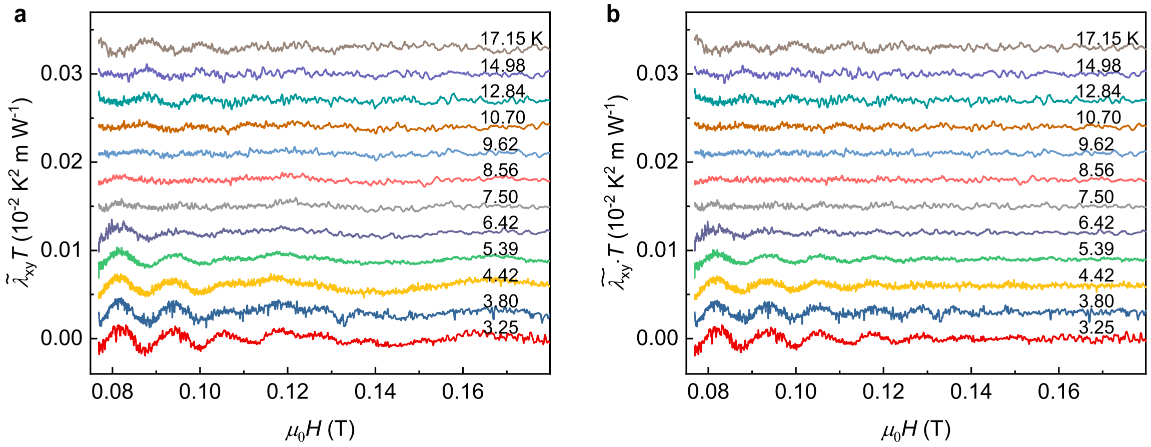

Supplementary Fig. 5. **High pass filter for $\Delta\lambda_{xy}T$.** Panel (a) shows the oscillation components $\Delta\lambda_{xy}T$ after a fifth-order polynomial background subtraction from the raw data. Panel (b) shows the oscillation components $\Delta\lambda_{xy}T$ with the 75 T High Pass Filter (HPF). Source data are provided as a Source Data file.

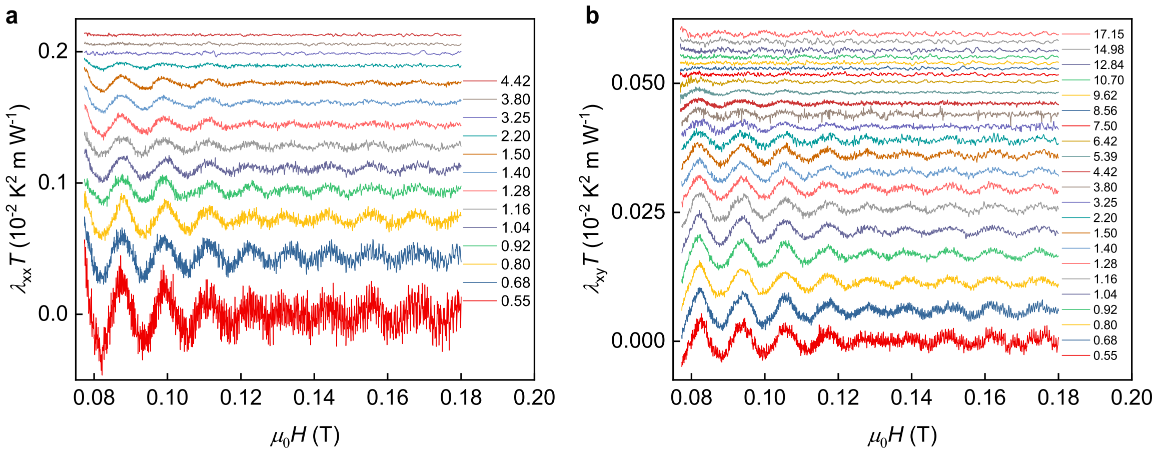

Supplementary Fig. 6. **The band pass filter for oscillations in both longitudinal and transverse thermal resistivities.** Panel (a) shows the oscillation components of the longitudinal thermal resistivity $\lambda_{xx}T$ with the 75 T High Pass Filter (HPF). Panel (b) shows the oscillation components of the thermal Hall resistivity $\lambda_{xy}T$ with the 75 T High Pass Filter (HPF). Source data are provided as a Source Data file.

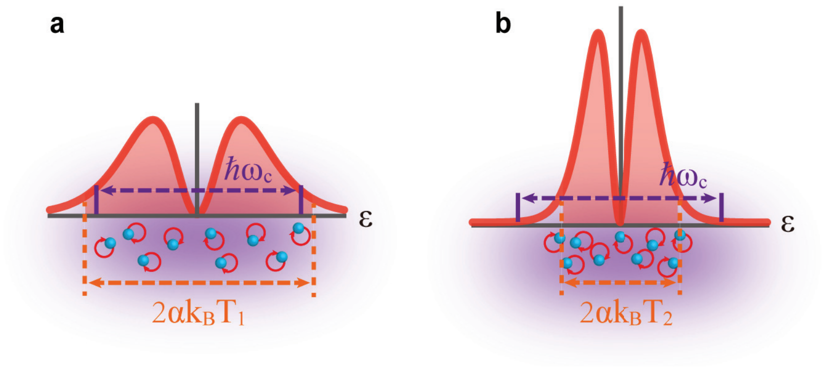

Supplementary Fig. 7. The equivalent energy dispersion $(\frac{\varepsilon-\varepsilon_\mathrm{F}}{k_BT})^2 D(\varepsilon)$ in semi-classical expression of electronic heat transport at T_1 (a) and T_2 (b), where $T_1 > T_2$. Here, α is set to be 6.09, and the value of $2\alpha k_B T$ is compared with $\hbar\omega_c$. Under this α, if $2\alpha k_B T = \hbar\omega_c$, the quantum oscillation exactly disappears due to destructive microscopic interference. For electronic charge transport, the dispersion is a single peak distribution function, while for heat transport it shows double peaks. Due to phase smearing effects, the quantum oscillation is the superposition of all the electron oscillations. Since the distribution function is nearly symmetric with respect to ε_F, the constructive quantum oscillation can only have 0 or π phase difference with the oscillation of electrons locating at ε_F. Panel (a) shows at higher *T*, the oscillation is dominated by the electrons with phase shift of π. Panel (b) shows at lower *T*, the oscillation is dominated by the electrons locating at ε_F.

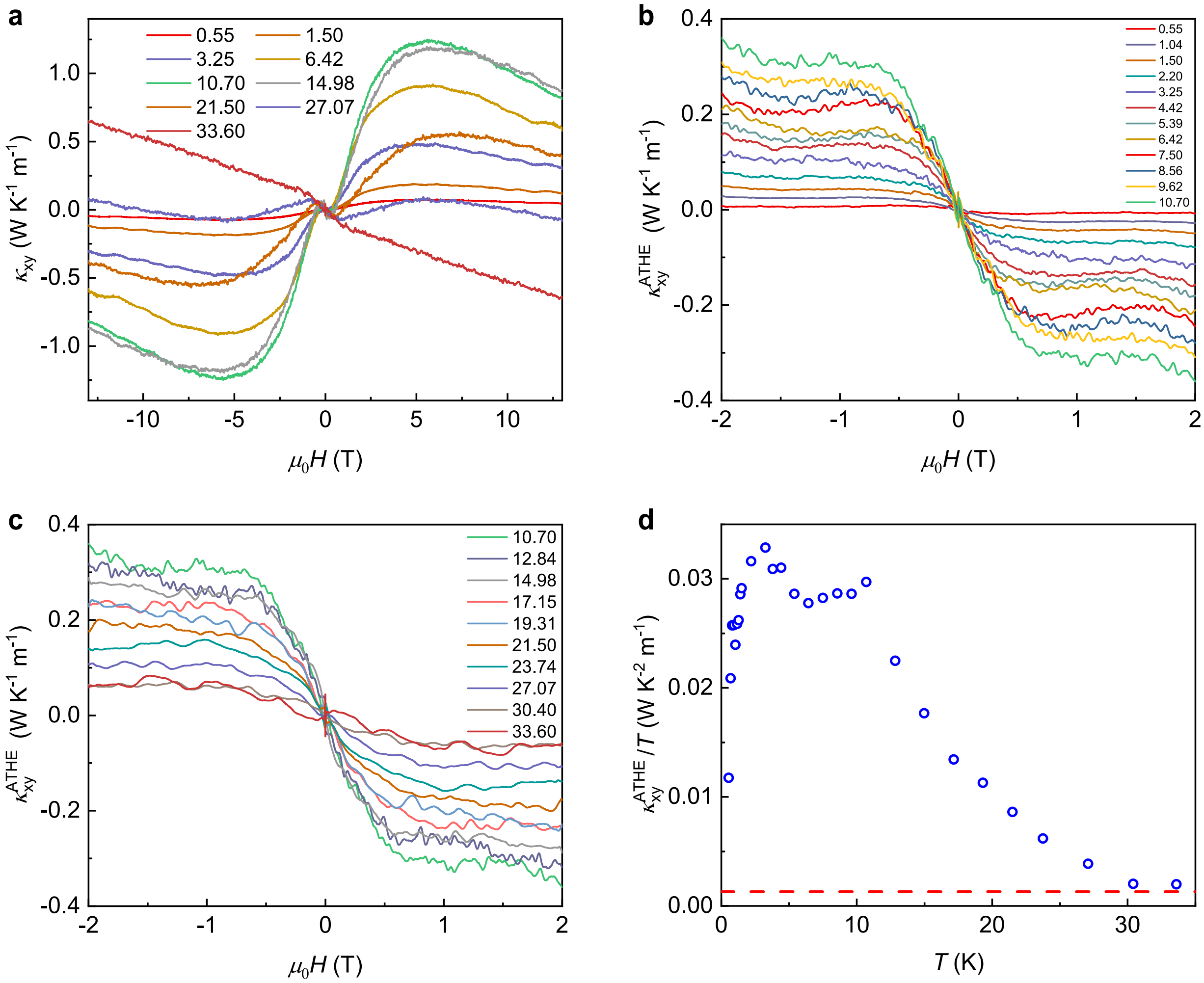

Supplementary Fig. 8. **Anomalous thermal Hall effect (ATHE) in CsV_3Sb_5.** Panel (a) shows the field dependence of the thermal Hall conductivity κ_{xy} measured in various temperatures. When the H is lower than ~ 2 T, an anomalous S shape shows up. Panel (b) and (c) show the extracted ATHE after subtracting the local linear background. Panel (d) shows the temperature evolution of the ATHE amplitude. The amplitude is obtained by averaging the data between the 1 T and 1.75 T field range. The red dashed line shows the intrinsic ATHE estimated using WF law and the predicted intrinsic AHE[7]. As the temperature increases, the ATHE gets closer to the red dashed line. Source data are provided as a Source Data file.

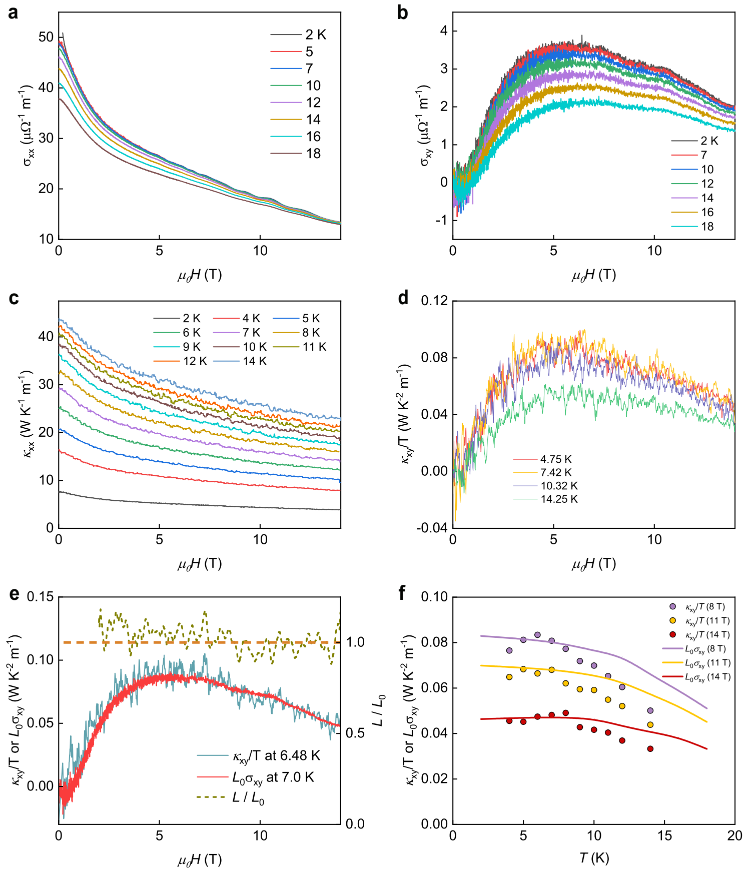

Supplementary Fig. 9. Longitudinal and transverse electrical and thermal conductivity measured in another CsV_3Sb_5 sample to verify the WF law od the non-oscillating background. The sample dimension is 1.7 mm × 0.5 mm × 0.06 mm. The longitudinal and transverse temperature differences were measured using the field-calibrated type-E thermocouple of 1-mil diameter in a Physical Properties Measurement System (PPMS) DynaCool from Quantum Design. Panel (a) and (b) show the field dependence of the electrical conductivity (a), and electrical Hall conductivity (b) measured at various temperatures with field H along c axis. Panel (c) and (d) show the field dependence of the thermal conductivity (c), and thermal Hall conductivity (d) measured using the thermocouples at various temperatures with field H along c axis. Panel (e) shows the comparison between the measured thermal Hall conductivity κ_{xy}/T (light blue curve) and electrical contribution to the thermal Hall conductivity $L_0\sigma_{xy}$ (red curve) at temperature T near 7.0 K. The dashed line shows the ratio of these two, defined as L/L_0, where L is the Wiedemann-Franz ratio. Panel (f) shows the temperature dependence of the measured thermal Hall conductivity κ_{xy}/T (the dots) and electrical contribution to the thermal Hall conductivity $L_0\sigma_{xy}$ (the lines) at selected mu_0H = 8, 11, and 14 T. At each magnetic field and temperature T below 9 K, the dots are quite near the lines, indicating the WF law is satisfied. Source data are provided as a Source Data file.

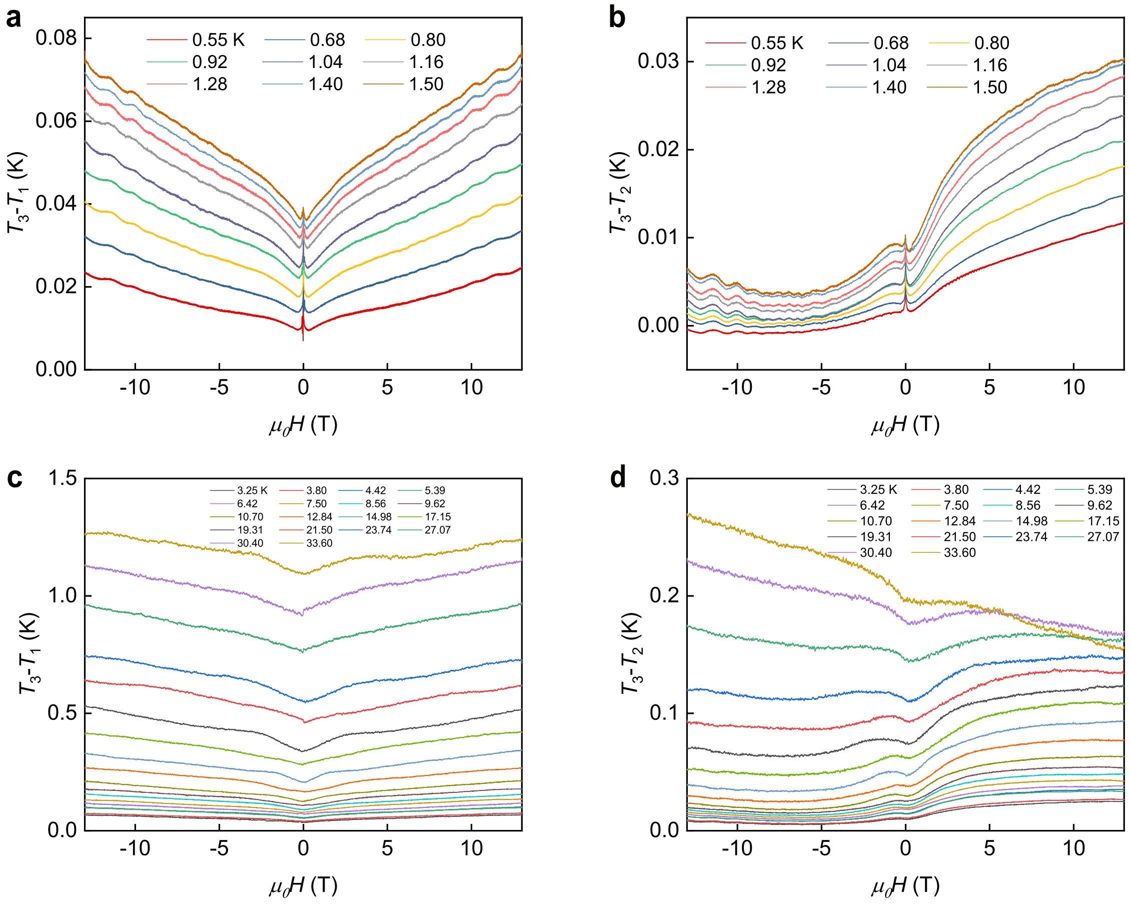

Supplementary Fig. 10. **Raw data of the thermometer readings in the thermal conductivity and thermal Hall effect measurements in CsV_3Sb_5.** Panel (a) and (b) show the longitudinal (a) and transverse (b) temperature differences measured on the sample at various T below 1.50 K. Panel (c) and (d) are the longitudinal (c) and transverse (d) temperature differences measured on the sample at higher T. Source data are provided as a Source Data file.

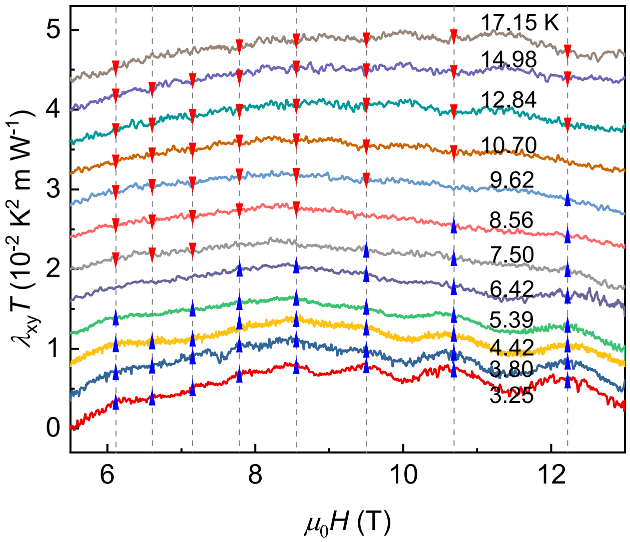

Supplementary Fig. 11. **Raw data of the quantum oscillation to clearly show the phase inversion in thermal Hall signal at different T ranging from 3.25 K to 17.15 K in CsV_3Sb_5.** Each curve is shifted with a constant. The red and blue arrows mark the phases of the oscillations. The blue arrows mark the peaks of the oscillations under low temperatures and specific magnetic fields, indicating that they are "in-phase." The red arrows mark the valleys of the oscillations under higher temperatures and the same magnetic field, indicating that the phase of the oscillations is inverted. Source data are provided as a Source Data file.

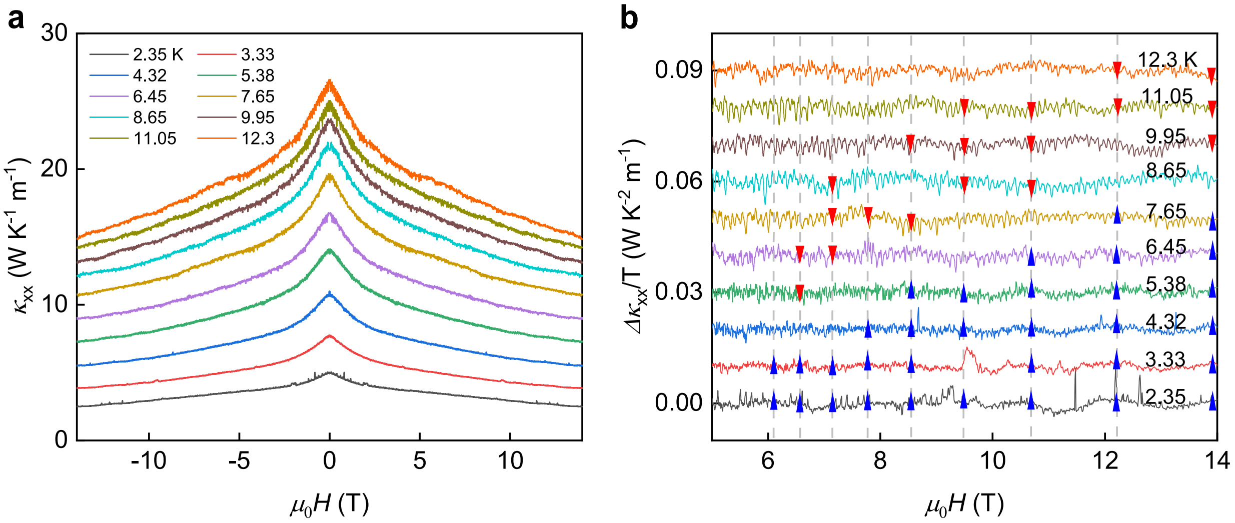

Supplementary Fig. 12. **The raw data of the thermal conductivity (Panel A) and the background-subtracted one (Panel B) showing the phase flip.** To analyze the 180-degree phase flip of quantum oscillation using κ_{xx}, we first extracted the oscillatory components of the δ orbit (87 T) using fifth-order polynomial background subtraction, and then we applied the 75 T high pass filter. The phase flip of quantum oscillations in κ_{xx} happens near $H = \frac{2\pi^2 k_B m^*}{1.62\mu_0 \hbar e} T$, which supports the phase flip in κ_{xy} as shown in Fig. 2d and Fig. S 11. Source data are provided as a Source Data file.

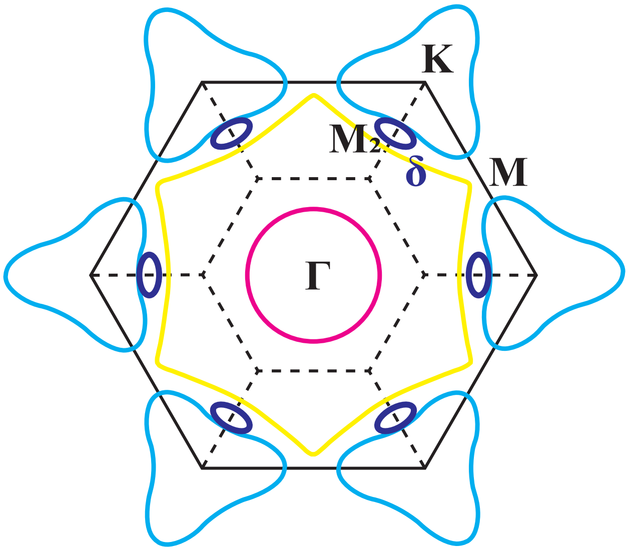

Supplementary Fig. 13. Scheme of the complex Fermi surface [8,9] of CsV_3Sb_5 at $k_Z = \frac{2}{3}\frac{\pi}{c}$. The black solid hexagon represents the Brillouin zone. The black dashed hexagons represent the reduced Brillouin zone in the $2a_0$ by $2a_0$ charge density wave phase. Larger Fermi surfaces with red, yellow, and light blue colors are consistent with previous reports[10,11]. The quantum oscillations measured from the thermal conductivity and thermal Hall contain four different orbits α, β, γ, δ whose frequencies are consistent with previous reports[10,11,12]. In particular, the δ orbit with dark blue color is located around the M_2 point of the reduced Brillouin zone, which arises from the interplay of Van Hove singularity and the CDW gap[9]. For these small quantum orbits α, β, and γ, they have not yet been identified in the photoemission measurements, their locations in the BZ are still unknown, and they are only theoretically investigated[13].

[1] Jaoui, A. *et al.* Formation of an Electron-Phonon Bifluid in Bulk Antimony. *Phys. Rev. X* **12**, 031023 (2022).

[2] Gan, Y. *et al.* Magneto-Seebeck effect and ambipolar Nernst effect in the CsV_3Sb_5 superconductor. *Phys. Rev. B* **104**, L180508 (2021).

[3] Chen, D. *et al.* Anomalous thermoelectric effects and quantum oscillations in the kagome metal CsV_3Sb_5. *Phys. Rev. B.* **105**, L201109 (2021).

[4] Shoenberg, D. *Magnetic oscillations in metals* (Cambridge university press, 2009).

[5] Ziman, J. M. *Principles of the Theory of Solids* (Cambridge university press, 1972).

[6] Van Houten, H., Molenkamp, L., Beenakker, C. & Foxon, C. Thermo-electric properties of quantum point contacts. *Semicond. Sci. Technol.* **7**, B215 (1992).

[7] Yu, F. *et al.* Concurrence of anomalous Hall effect and charge density wave in a superconducting topological kagome metal. *Phys. Rev. B* **104**, L041103 (2021).

[8] Zhou, S. Wang, Z. Chern Fermi pocket, topological pair density wave, and charge-4e and charge-6e superconductivity in kagomé superconductors. *Nat. Commun.* **13**, 7288 (2022).

[9] Li, H. *et al.* Small Fermi pockets intertwined with charge stripes and pair density wave order in a kagome superconductor. *Phys. Rev. X* **13**, 031030 (2023).

[10] Fu, Y. *et al.* Quantum transport evidence of topological band structures of kagome superconductor CsV_3Sb_5. *Phys. Rev. Lett.* **127**, 207002 (2021).

[11] Ortiz, B. R. *et al.* Fermi surface mapping and the nature of charge-density-wave order in the kagome superconductor CsV_3Sb_5. *Phys. Rev. X* **11**, 041030(2021).

[12] Broyles, C. *et al.* Effect of the interlayer ordering on the Fermi surface of Kagome superconductor CsV_3Sb_5 revealed by quantum oscillations. *Phys. Rev. Lett.* **129**, 157001 (2023).

[13] Tan, H. *et al.* Emergent topological quantum orbits in the charge density wave phase of kagome metal CsV_3Sb_5. Preprint at https://arxiv.org/abs/2303.04924 (2023).
